# Cognitive abilities, insurance decisions, and labor supply behavior: evidence from rural China

**DOI:** 10.3389/fpubh.2024.1421600

**Published:** 2024-06-28

**Authors:** Ziyue Yang

**Affiliations:** Central University of Finance and Economic, School of Insurance, Beijing, China

**Keywords:** social insurance, labor supply, peer effect, cognitive abilities, income effect

## Abstract

**Introduction:**

How cognitive abilities affect financial and economic decision is an important issue that has attracted the attention of economics.

**Method:**

This paper uses the China Family Panel Studies (CFPS) 2010, 2014, and 2018 survey data to empirically test the impact of cognitive skills on the insurance participation decisions in rural China.

**Results and discussion:**

The results show that higher word ability is correlated to higher social health insurance participation and both word and math ability leads to higher social pension participation. Mechanism analysis reveals that individuals with higher cognitive skills are more likely to be affected by peers in insurance decision, and higher cognitive skills increase personal income that enables them to enroll in the social insurance. Further investigation of labor supply behavior suggests that while cognitive skills positively affect non-agricultural labor participation, cognitive skills amplify the negative effect of social security on labor supply.

## 1 Introduction

The importance of cognitive abilities has been widely recognized for economic preferences (1) and economic decisions (2). Cognitive abilities also play a significant role in financial behaviors and decisions, such as risk aversion (3, 4) and financial investment (5).

Insurance decision is a crucial financial decision for households and individuals. Several studies have investigated the impact of cognitive skills on insurance decisions. Chatterjee and Nielsen (6) found that individuals with higher cognitive ability are more likely to purchase health insurance. McGarry et al. (7) found positive effect of cognitive ability on the purchase of long-term care insurance. Similar results are found for pension enrolment (8). However, little is known about whether and how do cognitive abilities affect insurance purchase in developing countries, especially in the Chinese context. This is of great importance, as China has an aging population but low insurance enrolment rate. Zhang and Li (9) are among the few to study this topic and found that cognitive ability improves health insurance participation in China.

This paper examines the correlation between cognitive skills and the enrolment in the New Rural Cooperative Medical Scheme and the New Rural Pension Scheme, which are the largest social health insurance and social pension scheme in rural China. This paper finds that word ability significantly improves the likelihood of having health insurance while both word and math ability enhances the purchase of social pension, indicating the different role of different abilities in financial decisions. This paper further investigates two potential mechanisms underlying the positive impact of cognitive ability on insurance decision, namely, peer effect and income effect. It is found that individuals with higher cognitive ability are more likely to be affected by peers in insurance purchase decisions, which is in line with knowledge transmission and learning behavior in the peer effects (10, 11). It is also suggested that higher cognitive ability is correlated with higher personal income, so as to improve insurance purchase ability, supporting an income effect.

This paper goes beyond insurance decision and looks into economic decision of rural residents by further investigating the impact of cognitive skills on labor supply behavior. Cognitive abilities have strong implications for labor supply behavior among other labor market behaviors and outcomes (12, 13). This paper examines the impact of cognitive abilities on non-agricultural work for rural residents in China and finds that higher cognitive abilities lead individuals to participate more in non-agricultural work and work longer in non-agricultural job.

The mediation role of cognitive skills in the effect of social insurance on labor supply is also investigated. The relationship between social security and labor supply has long been of great interest for economists (14–17). Studies tend to suggest a negative effect of social insurance on the labor supply (15, 18–21). Little is known about the heterogenous effect of social insurance on labor supply when individuals are of different cognitive abilities. This paper investigates the mediation role of cognitive abilities and finds that cognitive ability enhances the effect of social insurance on labor supply, i.e., the negative effect on labor supply is more pronounced among higher cognitive individuals.

This study contributes to the literature in the following ways. First, this paper provides new empirical evidence for the literature of the impact of cognitive skills on an important financial decision, insurance purchase, in the developing economy. This study focuses on the rural residents in China where the insurance coverage is relatively insufficient. Second, this paper investigates the peer effect and income effect which are the two potential mechanisms underlying the impact of cognitive skills on financial decision, which helps to better understand how cognitive skills impact individual's information process and economic decisions. Third, this paper goes beyond financial decision by focusing on labor supply behavior. This study examines the effect of cognitive skills on labor supply in rural China, which is of great importance considering China is facing severe aging and population reduction. This study also investigates the mediation role of cognitive skills on the effect of social security on labor supply, which contributes to the literature of determinants of labor supply.

The rest of the paper is organized as follows: in Section 2 the institutional background of social health insurance and social pension in rural China is introduced; Section 3 introduces the data used in this study, the variables construction and descriptive statistics; Section 4 shows and discusses on the main regressions, and further investigate the underlying mechanisms; Section 5 includes further analysis on labor supply behavior; Section 6 provides with several heterogeneity analysis results; and the Section 7 summarizes the paper.

## 2 Institutional background

### 2.1 New rural cooperative medical scheme

China's basic medical insurance system consists of three parts: basic medical insurance for urban employees, basic medical insurance for urban residents and new rural cooperative medication scheme (NCMS hereafter). The three target groups were different. Basic medical insurance coverage for urban employees is for individuals working for urban employers, and basic medical insurance coverage for urban residents is for minors who do not have basic medical insurance for urban employees and who do not have a job. The coverage of the new rural cooperative medical scheme is for all rural residents. The new rural cooperative medication policy was gradually implemented nationwide beginning in 2003, with government coordination and voluntary participation from farmers (22); since then, it has been rapidly promoted. At the end of 2008, the new rural cooperation medication policy basically achieved full coverage at the county level (23). In 2016, the State Council decided to integrate the new rural cooperation medication policy and basic medical insurance for urban residents into basic medical insurance for urban and rural residents (24). The health insurance is found to benefit residents in several ways (25, 26).

### 2.2 New rural pension scheme

China started the new rural pension scheme (NRPS hereafter), a voluntary public pension program, to the rural population in 2009. By 2012, the NRPS covered nearly all the counties in china (27). Participation in the NRPS is voluntary. Local rural residents aged 16 and above are qualified for the NRPS if they are not students at school, or are not insured by the urban pension scheme. NRPS participants aged 16–60 need to make a minimum 100 Yuan annual contribution in RMB to their individual accounts, while enrollees aged 60 and above need not. These individual accounts are fully funded, and contributions come from enrollees, the village, and the local government, which subsidizes 30 yuan for the individuals' annual contribution. Upon reaching age 60, qualified NRPS participants can receive a basic pension benefit of 55 yuan per month, which is entirely subsidized by the government, and a monthly distribution from their individual accounts. individuals below the age of 45 at the NRPS roll-out must contribute for at least 15 years to qualify for NRPS payments, while those aged 45–59 are required to pay annual premiums continuously until age 60 to receive payments. Enrollees aged 60 and above can directly receive the basic pension benefit as long as at least one of their adult children is enrolled in the NRPS. This family binding policy is to broaden the coverage of the pension scheme and to encourage the enrollment of adult children. individuals aged 60 and above are automatically eligible for the basic pension benefit if they have no adult children. In sum, during both the accumulation and decumulation phases, NRPS participants enjoy substantial government subsidies, and older adult enrollees are rewarded the most.

## 3 Data and variable

### 3.1 Data

The main dataset in this paper is drawn from three waves of China Family Panel Studies (CFPS), 2010, 2014 and 2018 survey. The CFPS survey is a nationally representative, longitudinal survey of Chinese communities, families, and individuals initially launched in 2010 by the Institute of Social Science Survey (ISSS) of Peking University. The nationwide CFPS baseline survey in 2010 successfully interviewed 14,798 households from 635 communities, including 33,600 adults and 8,990 children, in 25 designated provinces, with an approximate response rate of 81%, and the majority of non-responses were due to non-contact (28). The stratified multistage sampling strategy ensures that the CFPS sample represents 95% of the total population in 2010 (29).

In order to enlarge the sample size, we combined the three waves of the CFPS (2010, 2014, and 2018) into pooled cross-sectional data. These three waves of the CFPS survey have the same questionnaire on the cognitive ability of adult interviewees. In this paper, the insurance participation decision and labor supply behavior are studied, so I use the sample of adult individuals in rural China aged 25 to 55, who are still in the labor market. Then I match the sample data with family and parental information and drop the observations with missing values for relevant variables. As the CFPS investigates all household family members, the parents and children could be matched completely. Eventually a sample of xx rural individuals with complete household and parental information is formed.

### 3.2 Main variables

#### 3.2.1 Cognitive skills

The CFPS 2010, 2014 and 2018 has two tests to measure cognitive ability, i.e., the word test and math tests, and both are designed to be taken by both adults and children aged 10–15 years with the same rules and questions. The test questions and scores are comparable for the three survey years.

The word test measures the ability to recognize and pronounce words in Mandarin. It consists of eight sheets, and each sheet has 34 words or phrases. The math test measures cognitive ability in mathematics and logic analysis. The math test contains four problem sets with 24 questions in each set. In the estimation, age-standardized scores rather than raw scores are used for both the language and math tests.^1^ Specifically, the age-standardized word test score of an individual equals the raw word test score minus the average word test score of the sample individuals with same age, divided by their standard deviation of the word test scores. The same approach is adopted for calculating the age-standardized math test score. Two variables are constructed accordingly, *Word_test* and *Math_test*.

#### 3.2.2 Insurance participation

In this paper I use whether the sample individual participated in the social health insurance and whether the sample individual participated in the social pension scheme to measure the insurance participate behavior. In rural China, residents can choose whether to participate in the New Cooperative Medical Scheme (NCMS) which is the largest social health insurance scheme for residents of rural hukou, and can choose whether to participate in the New Rural Pension Scheme (NRPS) which is the largest social pension scheme for residents of rural hukou.

Two variables are constructed according, *NCMS* equals one if the individual participated in the New Cooperative Medical Scheme in the survey year, 0 otherwise. *NRPS* equals one if the individual participated in the New Rural Pension Scheme in the survey year, 0 otherwise. In this paper I did not use the participation in the commercial insurance because the enrolment rate of commercial insurance is extremely low in rural China which is below 5 percent.

#### 3.2.3 Labor supply behavior

The purpose of this study was to investigate the impact of cognitive skills on insurance participation and labor supply. This study mainly examined the participation of women in non-agricultural labor. Two variables were constructed to examine the participation of rural residents in non-agricultural labor in this study. First, whether the sample individual participated in non-agricultural labor, *Nonagri work* was determined. If the rural individual participated in work other than agriculture, including their own business and employed work, they were considered to be involved in non-agricultural labor. Second, the number of non-agricultural working hours, *Nonagri hours*, were measured. In the past year, the average number of non-agricultural working hours per week was calculated; if the women did not perform non-agricultural labor, then the number of non-agricultural labor hours was 0. In the regression analysis, I use the logarithm of non-agricultural labor hours plus 1.

Due to the fact that agricultural labor is hard to arrange, rural families often find it difficult to freely choose whether to engage in agricultural labor, and the duration of agricultural labor is also affected by the objective attributes of agricultural labor; therefore, it is difficult for agricultural labor status to truly reflect labor choices of individuals and households. Therefore, this study did not mainly focus on the participation in agricultural work.^2^

### 3.3 Descriptive statistics

The descriptive statistics of the main variables used for the empirical analysis in this paper are listed in Table 1. As seen in the table, among the rural residents in the sample of this study, the average participation rate of social health insurance is 85.7%, which is high, considering that the sample included in this study came from surveys conducted in 2010 and subsequent years, and the proportion of insured individuals was consistent with the statistical results of previous studies. The proportion of having social pension is 37.6% and is much lower than the participation rate of health insurance. On average, about 42% of sample rural residents are involved in non-agricultural work and the average weekly number of hours spent in non-agricultural labor is approximately 21 h.

**Table 1:** The main variable descriptive statistics.

**Variable**	**Observation value**	**Mean value**	**Standard deviation**	**Minimum value**	**Median value**	**Maximum value**
**Dependent variables**						
NCMS	14,412	0.857	0.350	0	1	1
NRPS	14,412	0.376	0.484	0	0	1
Non-agricultural labor participation	13,874	0.421	0.494	0	0	1
Non-agricultural working hours	13,625	21.04	29.21	0	0	168
**Independent variables**						
Word test (Raw score)	14,412	18.90	9.327	0	21	34
Math test (Raw score)	14,412	10.05	5.402	0	12	24
Gender	14,412	0.513	0.500	0	1	1
Years of education	14,412	7.433	3.770	0	9	19
Age	14,412	38.94	8.188	25	39	55
Married	14,412	0.971	0.166	0	1	1
Self-assessment of health	14,412	3.581	1.232	1	4	5
Chronic disease	14,412	0.107	0.309	0	0	1
Household per capita income	14,412	12,705	23,759	0	8,333	1.690e+06
Family size	14,412	4.802	1.849	1	5	26
Father's age	14,412	67.24	10.57	39	67	113
Father's education stage	14,412	2.017	1.144	1	2	9
Mother's age	14,412	65.61	10.07	39	65	146
Mother's education stage	14,412	1.830	1.420	1	1	9
Spouse's age	14,412	48.19	14.67	16	47	93
Spouse education stage	14,412	2.452	1.353	1	2	9

The raw scores of word test and math test are 18.9 (full scores of 34) and 10 (full scores of 24) respectively. From the perspective of individual characteristics, about 51% of the sample is male, the average age of the rural residents in the sample was 38.9 years, the average number of years of education was 7.4 years, more than 97% of the sample was married, and 10.7% of the sample had chronic diseases. From the perspective of family characteristics, the average per capita annual income of the family in the sample was close to 12700 yuan, and the average number of family members was 4.8 people. The spouse and parental characteristics of the sample are also included in the Table 1.

## 4 Main results

### 4.1 Empirical strategy

The main research issue of this study was the impact of cognitive skills on the insurance participation and labor supply behavior. To analyse this problem, the following ordinary least squares (OLS) model was used in this paper:


$$\begin{array}{l} NCMS_{ipt}/NRPS_{ipt}=\alpha +\beta Cognition_{ipt}+\gamma x_{ipt}+Province_{p}\\ \;+\;Survey\_year_{t}+\varepsilon_{ipt} \end{array}$$

where *NCMS*_*ipt*_/*NRPS*_*ipt*_ is whether individual *i* from province *p* in the survey year *t* has participated in the NCMS or the NRPS insurance. *Cognition*_*ipt*_ is the cognitive skills of the individual, which contains two variables, *Word*_*test*_*ipt*_ and *Math*_*test*_*ipt*_ measuring the word cognitive ability and math cognitive ability respectively. Coefficient β is an estimate of the impact of cognitive skills on the insurance participation, which was one of the main focuses of this study; **x**_**ipt**_ is a series of variables, such as personal characteristics, family characteristics, spouse and parental characteristics, including age, education level, marital status, self-assessed health level, chronic diseases, family per capita income, family size, parental and maternal age, education level, spousal age, education level and other variables, related to whether an individual participates in health insurance and may affect women's labor supply decision-making. Finally, this study evaluated the fixed effect of the province and the fixed effect of the survey year; and ε_*ipt*_ indicates the disturbance item used to control for the impact of unobservable factors in the province and the survey year.

### 4.2 Benchmark results

Table 2 shows the regression results of cognitive skills on the participation in the New Cooperative Medical Scheme. Column (1) and Column (3) are the results of raw word test score and math test score, while Column (2) and (4) are based on the age-standardized cognitive skills. Taking Column (2) as an example to illustrate, the coefficient of *Word_test* 0.011 indicates that an increase by one standard error of word test enhances the probability of NCMS by 1.1 percent. However, math test does not play a significant role in the participation of NCMS from Columns (3) and (4).

**Table 2:** Cognitive skills and the participation in the New Cooperative Medical Scheme.

	**(1)**	**(2)**	**(3)**	**(4)**
	**Dependent variables**			
**Independent variables**	***NCMS*** **(Raw score)**	***NCMS*** **(Standardized)**	***NCMS*** **(Raw score)**	***NCMS*** **(Standardized)**
Word test	0.001^***^	0.012^***^		
	(0.000)	(0.004)		
Math test			0.000	0.002
			(0.001)	(0.005)
Gender	0.012^*^	0.012^**^	0.012^**^	0.012^**^
	(0.006)	(0.006)	(0.006)	(0.006)
Education	−0.008^***^	−0.008^***^	−0.007^***^	−0.006^***^
	(0.001)	(0.001)	(0.001)	(0.001)
Age	0.003^***^	0.002^***^	0.003^***^	0.002^***^
	(0.001)	(0.001)	(0.001)	(0.001)
Ethnicity	0.013	0.013	0.014	0.014
	(0.011)	(0.011)	(0.011)	(0.011)
Number of children	0.017^***^	0.017^***^	0.016^***^	0.016^***^
	(0.004)	(0.004)	(0.004)	(0.004)
Marital status	0.033	0.034	0.035	0.035
	(0.022)	(0.022)	(0.022)	(0.022)
Self-assessment of health	−0.001	−0.001	−0.001	−0.001
	(0.003)	(0.003)	(0.003)	(0.003)
Chronic disease	0.008	0.008	0.008	0.008
	(0.009)	(0.009)	(0.009)	(0.009)
Family characteristic	YES	YES	YES	YES
Parental characteristics	YES	YES	YES	YES
Spousal characteristics	YES	YES	YES	YES
Provincial fixed effect	YES	YES	YES	YES
Survey year fixed effect	YES	YES	YES	YES
Observations	14,412	14,412	14,412	14,412
R^2^	0.142	0.142	0.141	0.141

The robust standard errors of heteroscedasticity are in parentheses. In Columns (1) and (3) the word and math test scores are raw test scores, while in Columns (2) and (4) they are age-standardized test scores. The family characteristic control variables include family per capita income and family size. The parental characteristics control variables included the following: father's age, father's education, mother's age, and mother's education. The control variables for spousal characteristics included spousal age and spousal education. ^*^p <0.1; ^**^p <0.05; ^***^p <0.01.

Judging from the results for the other variables, males have higher probability of purchasing health insurance, being older, having lower education, being married all have positive impacts on the probability of purchasing health insurance.

Table 3 shows the regression results of cognitive skills on the participation in the New Rural Pension Scheme. Column (1) and Column (3) are the results of raw word test score and math test score, while Column (2) and (4) are based on the age-standardized cognitive skills. Taking Column (2) as an example to illustrate, the coefficient of *Word_test* 0.026 indicates that an increase by one standard error of word test enhances the probability of enrolling in the NRPS by 2.6 percent. Similarly, it can be seen from Column (4) that an increase by one standard error of math test enhances the probability of having NRPS by 2.6 percent. Meanwhile, being older and being in lower self-rated health status have negative impacts on the NRPS participation. I conduct a robustness check by using the average age-standardized cognitive scores of word and math test, and the results are presented in Appendix Table 1. The results are robustness to different measurement of cognitive scores.

**Table 3:** Cognitive skills and the participation in the new rural pension scheme.

	**(1)**	**(2)**	**(3)**	**(4)**
	**Dependent variables**			
**Independent variables**	***NRPS*** **(Raw score)**	***NRPS*** **(Standardized)**	***NRPS*** **(Raw score)**	***NRPS*** **(Standardized)**
Word test	0.003^***^	0.026^***^		
	(0.001)	(0.005)		
Math test			0.003^**^	0.015^**^
			(0.001)	(0.006)
Gender	0.011	0.012	0.011	0.011
	(0.008)	(0.008)	(0.008)	(0.008)
Education	−0.001	−0.001	0.001	0.001
	(0.001)	(0.001)	(0.002)	(0.002)
Age	0.007^***^	0.006^***^	0.006^***^	0.006^***^
	(0.001)	(0.001)	(0.001)	(0.001)
Ethnicity	−0.005	−0.006	−0.003	−0.003
	(0.014)	(0.014)	(0.014)	(0.014)
Number of children	0.003	0.003	0.001	0.002
	(0.005)	(0.005)	(0.005)	(0.005)
Marital status	−0.005	−0.005	−0.002	−0.002
	(0.016)	(0.016)	(0.016)	(0.016)
Self-assessment of health	−0.010^***^	−0.010^***^	−0.011^***^	−0.011^***^
	(0.004)	(0.004)	(0.004)	(0.004)
Chronic disease	0.017	0.017	0.017	0.017
	(0.012)	(0.012)	(0.012)	(0.012)
Family characteristic	YES	YES	YES	YES
Parental characteristics	YES	YES	YES	YES
Spousal characteristics	YES	YES	YES	YES
Provincial fixed effect	YES	YES	YES	YES
Survey year fixed effect	YES	YES	YES	YES
Observations	14,412	14,412	14,412	14,412
R^2^	0.250	0.250	0.249	0.249

The robust standard errors of heteroscedasticity are in parentheses. In Columns (1) and (3) the word and math test scores are raw test scores, while in Columns (2) and (4) they are age-standardized test scores. The family characteristic control variables include family per capita income and family size. The parental characteristics control variables included the following: father's age, father's education, mother's age, and mother's education. The control variables for spousal characteristics included spousal age and spousal education. ^*^p <0.1; ^**^p <0.05; ^***^p <0.01.

### 4.3 Mechanism analysis

#### 4.3.1 Peer effect on insurance enrolment

Researchers have long been interested in the peer effects in program participation (10) and in financial decisions such as saving, investment, and insurance purchase (30, 31). In the Chinese context, Liu et al. (11) found significant peer effects in the health insurance purchase decision.

As the main mechanism underlying peer effect in financial decision is through knowledge transmission and learning (10, 11), individuals with higher cognitive ability are more likely to be affected by peers. As a result, those who have higher cognitive ability are more likely to take up social insurance decision. In order to examine the mechanism of peer effect, I estimate the following equation:


$$\begin{array}{l} NCMS_{ipt}/NRPS_{ipt}=\alpha +\beta_{1}Cognition_{ipt}*INS\_ratio_{ipt}\\ \;+\;\beta_{2}Cognition_{ipt}+\beta_{3}INS\_ratio_{ipt}+\gamma x_{ipt}\\ \;+\;Province_{p}+Survey\_year_{t}+\varepsilon_{ipt} \end{array}$$

where *NCMS*_*ipt*_/*NRPS*_*ipt*_ is whether individual *i* from province *p* in survey year *t* has participated in the NCMS or the NRPS insurance. *Cognition*_*ipt*_ is the cognitive skills of the individual, which contains two variables, *Word*_*test*_*ipt*_ and *Math*_*test*_*ipt*_ measuring the word cognitive ability and math cognitive ability respectively. *INS*_*ratio* is the proportion of residents in the same counties who participate in the social insurances. The coefficient of *INS*_*ratio* indicates the peer effect in the insurance purchase decision. We mainly focus on the coefficient of the interaction term between *INS*_*ratio* and *Cognition*_*ipt*_, β_1_, which captures the impact of cognitive skills on the peer effect in insurance purchase decision. If β_1_ is positive, it means that individuals with higher cognitive ability are more likely to be affected by peers in making decision concerning insurance participation. Other variables are the same as in the main regression specification.

Table 4 shows the results of peer effect analysis. The first two columns (1) and (2) are the results in terms of NCMS participation. The coefficients of the interaction term between *word test*/*math_test* and *Ins_ratio* are both significantly positive, indicating that the positive effects of peers in insurance enrolment are more pronounced with higher cognitive ability, which supports the peer effect mechanism. The Columns (3) and (4) are the results with NRPS participation as dependent variable. The results are quantitatively similar as those in the first two columns indicating the robust results of peer effects.

**Table 4:** Cognitive skills and the insurance participation: channel of peer effect.

	**(1)**	**(2)**	**(3)**	**(4)**
	**Dependent variables**			
	***NCMS***		***NRPS***	
**Independent variables**	***Word_test***	***Math_test***	***Word_test***	***Math_test***
Word/Math test^*^	0.011^***^	0.014^***^	0.012^***^	0.011^***^
INS_ratio	(0.003)	(0.003)	(0.003)	(0.003)
INS_ratio	0.040^***^	0.040^***^	0.007^***^	0.008^***^
	(0.003)	(0.003)	(0.003)	(0.003)
Word/Math test	−0.040^***^	−0.056^***^	−0.023^*^	−0.032^**^
	(0.012)	(0.013)	(0.012)	(0.014)
Gender	0.010	0.010	0.009	0.009
	(0.007)	(0.007)	(0.008)	(0.008)
Education	−0.003^**^	−0.002	−0.001	0.002
	(0.001)	(0.001)	(0.001)	(0.002)
Age	0.003^***^	0.003^***^	0.005^***^	0.005^***^
	(0.001)	(0.001)	(0.001)	(0.001)
Ethnicity	0.006	0.006	−0.019	−0.016
	(0.015)	(0.015)	(0.015)	(0.015)
Number of children	0.013^***^	0.013^***^	0.002	−0.000
	(0.005)	(0.005)	(0.006)	(0.006)
Marital status	0.044^*^	0.044^**^	0.013	0.016
	(0.023)	(0.022)	(0.015)	(0.015)
Self-assessment of health	0.002	0.002	−0.012^***^	−0.012^***^
	(0.003)	(0.003)	(0.004)	(0.004)
Chronic disease	0.013	0.013	0.004	0.004
	(0.010)	(0.010)	(0.014)	(0.014)
Family characteristic	YES	YES	YES	YES
Parental characteristics	YES	YES	YES	YES
Spousal characteristics	YES	YES	YES	YES
Provincial fixed effect	YES	YES	YES	YES
Survey year fixed effect	YES	YES	YES	YES
Observations	9,981	9,981	9,981	9,981
R^2^	0.173	0.174	0.303	0.302

The robust standard errors of heteroscedasticity are in parentheses. All the test scores are age-standardized test scores. The family characteristic control variables include family per capita income and family size. The parental characteristics control variables included the following: father's age, father's education, mother's age, and mother's education. The control variables for spousal characteristics included spousal age and spousal education. ^*^p <0.1; ^**^p <0.05; ^***^p <0.01.

#### 4.3.2 Income effect

The second mechanism underlying the effect of cognitive skills on the insurance participation is the income effect. Higher cognitive skills are correlated with higher income. The effects of income on insurance enrolment are two-fold: one is the income effect indicating that higher income enables individuals to have more insurance coverage; the other is substitute effect indicating that higher income encourages individual to have more risk retention. As the premium of social insurance in rural China is relatively low and rural residents are mostly under-covered in terms of insurance, it is assumed that income effect dominants the substitute effect. In order to examine the income effect, I use personal annual income to measure income and estimate the following equation:


$$\begin{array}{l} Income_{ipt}=\alpha +\beta Cognition_{ipt}+\gamma x_{ipt}+Province_{p}\\ \;+\;Survey\_year_{t}+\varepsilon_{ipt} \end{array}$$

where *Income*_*i*_ is the annual personal income of individual *i*. *Cognition*_*i*_ is the cognitive skills of individual *i*, which contains two variables, *Word*_*test*_*ipt*_ and *Math*_*test*_*ipt*_ measuring the word cognitive ability and math cognitive ability respectively. Other variables are similar as in the main regression. Coefficient β is an estimate of the impact of cognitive skills on the income, which examines the income effect.

Table 5 shows the results of income effect analysis. From the first two columns, it can be seen that both word test and math test significantly improves personal income, which supports the channel of income. In terms of other control variables, being male, having higher education, being younger, married, and having better health all improve personal income. I also use household income per capita as an alternative measurement of income, and the results in Columns (3) and (4) are similar to personal income.

**Table 5:** Cognitive skills and the insurance participation: channel of income and education.

	**(1)**	**(2)**	**(3)**	**(4)**	**(5)**	**(6)**
	**Dependent variables**					
	**Personal income**		**Household income per capita**		**Years of schooling**	
**Independent variables**	***Word_test***	***Math_test***	***Word_test***	***Math_test***	***Word_test***	***Math_test***
Word/Math test	0.262^***^	0.281^***^	0.083^***^	0.089^***^	2.149^***^	2.903^***^
	(0.047)	(0.059)	(0.011)	(0.015)	(0.027)	(0.027)
Gender	2.002^***^	1.992^***^	−0.027	−0.031^*^	0.733^***^	0.338^***^
	(0.073)	(0.073)	(0.017)	(0.017)	(0.048)	(0.040)
Education	0.069^***^	0.057^***^	0.034^***^	0.031^***^	−0.076^***^	−0.104^***^
	(0.013)	(0.015)	(0.003)	(0.004)	(0.007)	(0.006)
Age	−0.045^***^	−0.047^***^	0.007^***^	0.007^***^	0.385^***^	0.229^***^
	(0.009)	(0.009)	(0.002)	(0.002)	(0.089)	(0.078)
Ethnicity	0.359^**^	0.367^**^	0.011	0.014	−0.248^***^	−0.199^***^
	(0.153)	(0.153)	(0.034)	(0.034)	(0.034)	(0.029)
Number of children	−0.021	−0.030	−0.094^***^	−0.097^***^	0.255^*^	0.332^***^
	(0.049)	(0.049)	(0.012)	(0.012)	(0.154)	(0.126)
Marital status	0.290^*^	0.318^*^	0.273^***^	0.282^***^	0.102^***^	0.054^***^
	(0.171)	(0.172)	(0.056)	(0.056)	(0.023)	(0.019)
Self-assessment of health	0.086^**^	0.082^**^	0.033^***^	0.032^***^	−0.072	−0.025
	(0.035)	(0.035)	(0.008)	(0.008)	(0.079)	(0.067)
Chronic disease	−0.061	−0.059	0.015	0.016	2.149^***^	2.903^***^
	(0.116)	(0.116)	(0.026)	(0.026)	(0.027)	(0.027)
Family characteristic	YES	YES	YES	YES	YES	YES
Parental characteristics	YES	YES	YES	YES	YES	YES
Spousal characteristics	YES	YES	YES	YES	YES	YES
Provincial fixed effect	YES	YES	YES	YES	YES	YES
Survey year fixed effect	YES	YES	YES	YES	YES	YES
Observations	12,317	12,317	14,412	14,412	14,412	14,412
R^2^	0.371	0.370	0.292	0.291	0.509	0.654

The robust standard errors of heteroscedasticity are in parentheses. All the test scores are age-standardized test scores. The family characteristic control variables include family per capita income and family size. The parental characteristics control variables included the following: father's age, father's education, mother's age, and mother's education. The control variables for spousal characteristics included spousal age and spousal education. ^*^p <0.1; ^**^p <0.05; ^***^p <0.01.

Finally, similar to the income effect, I checked whether higher cognitive skills lead to higher educational attainment as the education effect. The results using years of schooling as dependent variable are listed in Columns (5) and (6), indicating a significantly positive effect of cognitive skills on education.

## 5 Further analysis

### 5.1 Cognitive skills and the labor supply behavior

Cognitive skills not only affect insurance decisions, but also have important implications for labor market behaviors (12, 32), such as labor supply. In this paper, I further investigate whether and how do cognitive skills affect labor supply behavior in rural China. The following equation is estimated:


$$\begin{array}{l} Noagri\_work_{ipt}/Noagri\_hour_{ipt}=\alpha +\beta Cognition_{ipt}+\gamma x_{ipt}\\ \;+Province_{p}+Survey\_year_{t}+\varepsilon_{ipt} \end{array}$$

where *Noagri*_*work*_*ipt*_/*Noagri*_*hour*_*ipt*_ is the two measurements of labor supply, namely whether individual *i* participated in non-agricultural work, and the average hours participating in non-agricultural work. The two variables measure both extensive margin and intensive margin of labor supply. *Cognition*_*ipt*_ is the cognitive skills of individual *i*, which contains two variables, *Word*_*test*_*ipt*_ and *Math*_*test*_*ipt*_ measuring the word cognitive ability and math cognitive ability respectively. Other variables are the same as in the main specification. Coefficient β is an estimate of the impact of cognitive skills on the labor supply, which is of the most interest in this test.

Table 6 show the results of cognitive skills on the labor supply behavior. The first two Columns (1) and (2) are the results concerning non-agricultural work participation. It can be seen that one standard error of increase in word test and math test enhances the likelihood of participating in the non-agricultural work by 4.3% and 4.5% respectively among rural residents in China. Columns (3) and (4) are the results in terms of non-agricultural work hours. One standard error of increase in word test and math test enhances the hours spent in non-agricultural work by 1.4 and 1.6 respectively. The results prove that higher cognitive skills have positive impact on the labor supply in rural China. I also use agricultural labor hour as a robustness check and the results are presented in Appendix Table 2.

**Table 6:** Cognitive skills and the labor supply behavior.

	**(1)**	**(2)**	**(3)**	**(4)**
	**Dependent variables**			
	***Noagri work***		***Noagri hour***	
**Independent variables**	***Word_test***	***Math_test***	***Word_test***	***Math_test***
Word/Math test	0.041^***^	0.044^***^	0.135^***^	0.155^***^
	(0.005)	(0.006)	(0.020)	(0.025)
Gender	0.147^***^	0.146^***^	0.660^***^	0.653^***^
	(0.008)	(0.008)	(0.031)	(0.032)
Education	0.016^***^	0.014^***^	0.057^***^	0.050^***^
	(0.001)	(0.002)	(0.005)	(0.006)
Age	−0.008^***^	−0.009^***^	−0.032^***^	−0.033^***^
	(0.001)	(0.001)	(0.004)	(0.004)
Ethnicity	0.037^**^	0.038^**^	0.209^***^	0.212^***^
	(0.015)	(0.015)	(0.058)	(0.058)
Number of children	−0.021^***^	−0.023^***^	−0.063^***^	−0.068^***^
	(0.005)	(0.005)	(0.021)	(0.021)
Marital status	0.073^***^	0.077^***^	0.280^***^	0.293^***^
	(0.024)	(0.024)	(0.092)	(0.092)
Self-assessment of health	0.005	0.005	0.021	0.019
	(0.003)	(0.003)	(0.014)	(0.014)
Chronic disease	0.002	0.003	0.031	0.034
	(0.012)	(0.012)	(0.048)	(0.048)
Family characteristic	YES	YES	YES	YES
Parental characteristics	YES	YES	YES	YES
Spousal characteristics	YES	YES	YES	YES
Provincial fixed effect	YES	YES	YES	YES
Survey year fixed effect	YES	YES	YES	YES
Observations	13,874	13,874	13,625	13,625
R^2^	0.261	0.260	0.252	0.252

The robust standard errors of heteroscedasticity are in parentheses. All the test scores are age-standardized test scores. The family characteristic control variables include family per capita income and family size. The parental characteristics control variables included the following: father's age, father's education, mother's age, and mother's education. The control variables for spousal characteristics included spousal age and spousal education. ^*^p <0.1; ^**^p <0.05; ^***^p <0.01.

### 5.2 Cognitive skills, social insurance, and the labor supply behavior

The relationship between social security and labor supply has long been investigated (14–17). Many studies have found negative effects of social insurance on the labor supply, indicating a substitution effect, both in the developed economy and in the Chinese context. Blau and Goodstein (18) found that a small part of the decline in the labor supply in the United States from the 1960s to the 1980s was due to an increase in pension security levels, and the delay in retirement age and pension can explain a large part of the increase in the labor supply since the 1990s. Mastrobuoni (15) also found that the reduction of pension security has led to the increase in the average retirement age in the context of the United States. Oshio et al. (19) studied the impact of social security programs on the labor supply in Japan over the past 40 years and found that the decrease in social security levels brought about by social security reforms encouraged an increase in labor participation among older adult people. In research on China, Cheng (20) characterized the impact of the pension security system on labor decision-making through a theoretical model and used sample survey data from rural households for empirical analysis. The results showed that new rural pension system reduces the labor supply of rural residents. At the same time, insurance for urban workers and comprehensive insurance for famous agricultural workers encourage rural residents to engage in non-agricultural labor. The results showed the heterogeneity of the impact of different types of endowment insurance in China. Zhang (21), using health and pension tracking survey data from China, studied the impact of new agricultural insurance on the labor supply of rural older adult people using the breakpoint regression method. As a result, it was also found that the new agricultural insurance pension reduced the labor supply of older adult people.

However, little is known about how cognitive skills moderate the effect of insurance on labor supply. In this study, I further examine the moderate effect of cognitive skills by estimating the following equation:


$$\begin{array}{l} \;Noagri\_work_{ipt}/Noagri\_hour_{ipt}\\ =\alpha +\beta_{1}Cognition_{ipt}*INS_{ipt}+\beta_{2}Cognition_{ipt}+\beta_{3}INS_{ipt}\\ \;+\;\gamma x_{ipt}+Province_{p}+Survey\_year_{t}+\varepsilon_{ipt}\; \end{array}$$

where *Noagri*_*work*_*ipt*_/*Noagri*_*hour*_*ipt*_ is the two measurements of labor supply, namely whether individual *i* participated in non-agricultural work, and the average hours participating in non-agricultural work. *INS*_*ipt*_ is whether individual *i* has participated in the NCMS or the NRPS insurance. *Cognition*_*ipt*_ is the cognitive skills of individual *i*, which contains two variables, *Word*_*test*_*ipt*_ and *Math*_*test*_*ipt*_ measuring the word cognitive ability and math cognitive ability respectively. The coefficient of *INS* indicates the effect of having insurance coverage on labor supply. We mainly focus on the coefficient of the interaction term between *INS* and *Cognition*_*i*_, β_1_, which captures the moderate effect of cognitive skills on the impact of insurance on labor supply. Other variables are the same as in the main regression specification.

Table 7 presents the results of the specification above. Column (1) is the moderate effect of word test on the impact of NCMS on noagricultural work participation, while it is noagricultural work hour in Column (5). The other columns can be explained in the similar way. From the table we can see that the coefficients of INS are all significantly negative in eight regressions, indicating a substitute effect of social insurance on labor supply behavior. In terms of the interaction term, a significantly negative coefficient indicates that the substitute effect of social insurance is more pronounced when an individual has higher cognitive ability. All the interaction terms are significantly negative, supporting that cognitive ability enhances the effect of insurance in reducing labor supply.

**Table 7:** Cognitive skills, insurance participation and labor supply behavior.

	**(1)**	**(2)**	**(3)**	**(4)**
	**Dependent variables**			
	***Noagri work***			
**INS**=	***NCMS***		***NRPS***	
**Independent variables**	***Word_test***	***Math_test***	***Word_test***	***Math_test***
Word/Math test^*^	−0.028^***^	−0.021^**^	−0.012^*^	−0.014^*^
INS	(0.010)	(0.010)	(0.007)	(0.008)
Word/Math test	0.067^***^	0.062^***^	0.046^***^	0.049^***^
	(0.010)	(0.011)	(0.006)	(0.007)
INS	−0.110^***^	−0.108^***^	−0.025^***^	−0.024^***^
	(0.011)	(0.011)	(0.009)	(0.009)
Control variables	YES	YES	YES	YES
Observations	13,874	13,874	13,874	13,874
R^2^	0.267	0.265	0.262	0.261
	**(5)**	**(6)**	**(7)**	**(8)**
	**Dependent variables**			
	***Noagri hour***			
**INS**=	***NCMS***		***NRPS***	
**Independent variables**	***Word_test***	***Math_test***	***Word_test***	***Math_test***
Word/Math test^*^	−0.101^**^	−0.082^**^	−0.053^*^	−0.063^**^
INS	(0.042)	(0.042)	(0.029)	(0.031)
Word/Math test	0.228^***^	0.226^***^	0.158^***^	0.180^***^
	(0.043)	(0.044)	(0.022)	(0.028)
INS	−0.438^***^	−0.432^***^	−0.086^**^	−0.084^**^
	(0.044)	(0.044)	(0.036)	(0.036)
Control variables	YES	YES	YES	YES
Observations	13,625	13,625	13,625	13,625
R^2^	0.258	0.257	0.253	0.252

The robust standard errors of heteroscedasticity are in parentheses. All the test scores are age-standardized test scores. Controls variables include gender, education, age, ethnicity, number of children, marital status, self-assessment of health, chronic disease. The family characteristic control variables include family per capita income and family size. The parental characteristics control variables included the following: father's age, father's education, mother's age, and mother's education. The control variables for spousal characteristics included spousal age and spousal education. ^*^p <0.1; ^**^p <0.05; ^***^p <0.01.

## 6 Heterogeneity analysis

### 6.1 Gender heterogeneity

Gender gaps exist in financial decision (33) and in labor supply behavior (34). In this section, I look into the gender gap in the impacts of cognitive skills on insurance purchase as well as on labor supply behavior as I did in the main regressions. First I split the sample individual into two subgroups, namely the female sample and the male sample, and then repeat the regressions in the main analysis in the two subgroups.

Table 8 presents the results of gender heterogeneity. Panel A of Table 8 is the results using female sample, and Panel B is the male sample. In terms of insurance decision, it is quite interesting that only word test matters for female sample. One plausible reason is that female depends more on communication ability while male depends more on logical ability. Among male sample, cognitive skills matter only for the social pension instead of health insurance, indicating that individuals make insurance decision differently based on different risks they are facing.

**Table 8:** The impact of cognitive skills: gender heterogeneity.

**Panel A: Female sample**				
	**(1)**	**(2)**	**(3)**	**(4)**
	**Dependent variables**			
	***NCMS***		***NRPS***	
**Independent variables**	***Word_test***	***Math_test***	***Word_test***	***Math_test***
Word/Math test	0.015^***^	0.003	0.028^***^	0.004
	(0.005)	(0.007)	(0.007)	(0.009)
	**(5)**	**(6)**	**(7)**	**(8)**
	**Dependent variables**			
	***Noagri work***		***Noagri hour***	
**Independent variables**	***Word_test***	***Math_test***	***Word_test***	***Math_test***
Word/Math test	0.037^***^	0.065^***^	0.102^***^	0.203^***^
	(0.007)	(0.009)	(0.026)	(0.034)
**Panel B: Male sample**				
	**(1)**	**(2)**	**(3)**	**(4)**
	**Dependent variables**			
	***NCMS***		***NRPS***	
**Independent variables**	***Word_test***	***Math_test***	***Word_test***	***Math_test***
Word/Math test	0.008	−0.000	0.023^***^	0.025^***^
	(0.005)	(0.007)	(0.007)	(0.009)
	**(5)**	**(6)**	**(7)**	**(8)**
	**Dependent variables**			
	***Noagri work***		***Noagri hour***	
**Independent variables**	***Word_test***	***Math_test***	***Word_test***	***Math_test***
Word/Math test	0.048^***^	0.024^***^	0.191^***^	0.112^***^
	(0.007)	(0.009)	(0.030)	(0.037)

The robust standard errors of heteroscedasticity are in parentheses. All the test scores are age-standardized test scores. Controls variables include gender, education, age, ethnicity, number of children, marital status, self-assessment of health, chronic disease. The family characteristic control variables include family per capita income and family size. The parental characteristics control variables included the following: father's age, father's education, mother's age, and mother's education. The control variables for spousal characteristics included spousal age and spousal education. ^*^p <0.1; ^**^p <0.05; ^***^p <0.01.

As for the labor supply behavior, it is found that both word and math tests have significantly positive impact on men's and women's labor supply. However, the impact of math test on labor supply for women is more than twice as for men, which indicates that math ability is more important in improving nonagriculatural labor supply for rural women.

### 6.2 Regional heterogeneity

China has large regional disparities, with eastern provinces being generally more developed. I divide the sample into two groups based on the province where the sample individual lives: eastern provinces (more developed) and non-eastern provinces (less developed). The regressions in the main analysis are repeated in the two subgroups.

Table 9 shows the results of regional heterogeneity. Panel A of Table 9 is the results of eastern sample, and Panel B is the non-eastern sample. From the table we can see that the significant impacts of cognitive skills on insurance purchase only exist among non-eastern sample, indicating large regional gap in insurance purchase. However, the impact of cognitive skills on labor supply are similar among eastern and non-eastern provinces.

**Table 9:** The impact of cognitive skills: household income heterogeneity.

**Panel A: Low household income**				
	**(1)**	**(2)**	**(3)**	**(4)**
	**Dependent variables**			
	***NCMS***		***NRPS***	
**Independent variables**	***Word_test***	***Math_test***	***Word_test***	***Math_test***
Word/Math test	0.007	0.008	0.025^***^	0.010
	(0.005)	(0.006)	(0.006)	(0.008)
	**(5)**	**(6)**	**(7)**	**(8)**
	**Dependent variables**			
**INS**=	***Noagri work***		***Noagri hour***	
**Independent variables**	***Word_test***	***Math_test***	***Word_test***	***Math_test***
Word/Math test	0.036^***^	0.042^***^	0.118^***^	0.146^***^
	(0.006)	(0.008)	(0.024)	(0.031)
**Panel B: High household income**				
	**(1)**	**(2)**	**(3)**	**(4)**
	**Dependent variables**			
	***NCMS***		***NRPS***	
**Independent variables**	***Word_test***	***Math_test***	***Word_test***	***Math_test***
Word/Math test	0.013^**^	−0.005	0.024^***^	0.020^**^
	(0.006)	(0.007)	(0.008)	(0.010)
	**(5)**	**(6)**	**(7)**	**(8)**
	**Dependent variables**			
**INS**=	***Noagri work***		***Noagri hour***	
**Independent variables**	***Word_test***	***Math_test***	***Word_test***	***Math_test***
Word/Math test	0.034^***^	0.033^***^	0.108^***^	0.120^***^
	(0.008)	(0.010)	(0.032)	(0.040)

The robust standard errors of heteroscedasticity are in parentheses. All the test scores are age-standardized test scores. Controls variables include gender, education, age, ethnicity, number of children, marital status, self-assessment of health, chronic disease. The family characteristic control variables include family per capita income and family size. The parental characteristics control variables included the following: father's age, father's education, mother's age, and mother's education. The control variables for spousal characteristics included spousal age and spousal education. ^*^p <0.1; ^**^p <0.05; ^***^p <0.01.

### 6.3 Household income heterogeneity

The last heterogeneity test is based on household income. The sample is split into two groups based on the household income per capita: low household income and high household income. The regressions in the main analysis are repeated in the two subgroups.

Table 9 shows the results of household heterogeneity. Panel A of Table 9 is the results of low household sample, and Panel B is the high household sample. From the table we can see that the significant impacts of cognitive skills on insurance purchase are mainly for low household income sample, indicating a gap in insurance purchase for different household income. However, the impact of cognitive skills on labor supply are similar among individuals with low or high household income.

## 7 Conclusion

This study focuses on the impact of cognitive skills on the insurance purchase decisions in rural China using CFPS data from three waves (2010, 2014, and 2018). This study finds that rural residents who have higher word cognitive ability are more likely to participate in the social health insurance, the NCMS, and individuals with either higher word or math ability are more likely to participate in the social pension, the NRPS. The results suggest the different role of word and math ability in financial decision and different decisions in terms of health insurance and pension enrolment.

This study further investigates the mechanisms of cognitive skills on the insurance purchase behavior in rural China. The results show that individuals with higher cognitive ability are more likely to be affected by peers in making financial decision, which is in line with the knowledge transmission and learning effect. Besides, individuals with higher cognitive ability tend to have higher personal income, enabling them to participate more in social insurance.

The paper goes beyond financial decision by looking into the labor supply behavior of rural resident. The results suggest that individuals with higher cognitive skills are more likely to participate in non-agricultural work and tend to work longer in non-agricultural job. However, as a negative role of social insurance on the labor participation is suggested in the Chinese context, this paper finds that cognitive skills amplify this substitute effect, i.e., the negative effects of social insurance on labor supply are larger in the absolute value when the individual has higher cognitive skills.

Finally, this study finds that the effect of cognitive skills on insurance decisions and labor supply behaviors are heterogenous in terms of different gender, different regions, and different household income.

## Data availability statement

Publicly available datasets were analyzed in this study. This data can be found at: https://www.isss.pku.edu.cn/cfps/.

## Author contributions

ZY: Writing – original draft, Writing – review & editing.

## Footnotes

^1^One concern is the censoring of cognitive test scores due to the full mark. However, in our sample, only less than 1% of the observations achieve a full mark of either word or math test. Thus it is not a main concern in terms of the censoring of cognitive scores.

^2^I examine the impact on agricultural work as a robustness check and list the results in the Appendix Table.
