# Supplementary material for: Cognitive abilities, insurance decisions, and labor supply behavior: evidence from rural China
Source: Front Public Health. 2024 Jun 28;12:1421600. doi: 10.3389/fpubh.2024.1421600 (PMC11239384; doi:10.3389/fpubh.2024.1421600)
Supplement: Supplementary file 1 [file Table_1.docx]

Appendix

Appendix Table 1 Robustness check: average score of word and math test

|  | (1) | (2) | (3) | (4) |
| --- | --- | --- | --- | --- |
| Independent variables | Dependent variables | | | |
|  | *NCMS*  (Standardized) | *NRPS*  (Standardized) | *Noagri work* | *Noagri hour* |
| Word test | 0.011** | 0.031*** | 0.059*** | 0.201*** |
|  | (0.005) | (0.006) | (0.007) | (0.026) |
| Gender | 0.012* | 0.011 | 0.146*** | 0.654*** |
|  | (0.006) | (0.008) | (0.008) | (0.032) |
| Education | -0.008*** | -0.002 | 0.012*** | 0.044*** |
|  | (0.001) | (0.002) | (0.002) | (0.006) |
| Age | 0.002*** | 0.005*** | -0.009*** | -0.034*** |
|  | (0.001) | (0.001) | (0.001) | (0.004) |
| Ethnicity | 0.013 | -0.005 | 0.036** | 0.204*** |
|  | (0.011) | (0.014) | (0.015) | (0.058) |
| Number of children | 0.017*** | 0.003 | -0.021*** | -0.063*** |
|  | (0.004) | (0.005) | (0.005) | (0.021) |
| Marital status | 0.034 | -0.004 | 0.074*** | 0.283*** |
|  | (0.022) | (0.016) | (0.024) | (0.092) |
| Self-assessment of health | -0.001 | -0.010*** | 0.005 | 0.021 |
|  | (0.003) | (0.004) | (0.003) | (0.014) |
| Chronic disease | 0.008 | 0.017 | 0.003 | 0.032 |
|  | (0.009) | (0.012) | (0.012) | (0.048) |
| Family Characteristic | YES | YES | YES | YES |
| Parental Characteristics | YES | YES | YES | YES |
| Spousal Characteristics | YES | YES | YES | YES |
| Provincial fixed effect | YES | YES | YES | YES |
| Survey year fixed effect | YES | YES | YES | YES |
| Observations | 14,412 | 14,412 | 13,874 | 13,625 |
| R^2^ | 0.142 | 0.250 | 0.262 | 0.253 |

Note: The robust standard errors of heteroscedasticity are in parentheses. All the test scores are age-standardized test scores. The family characteristic control variables include family per capita income and family size. The parental characteristics control variables included the following: father's age, father's education, mother's age, and mother's education. The control variables for spousal characteristics included spousal age and spousal education.

* p<0.1; ** p<0.05; *** p<0.01.

Appendix Table 2 Cognitive skills and the agricultural labor hour

|  | (1) | (2) |
| --- | --- | --- |
|  | Dependent variables | |
|  | *Agricultural work hour* | |
| Independent variables | *Word_test* | *Math_test* |
| Word/Math test | 0.011 | 0.060*** |
|  | (0.015) | (0.020) |
| Gender | 0.405*** | 0.401*** |
|  | (0.025) | (0.025) |
| Education | 0.011*** | 0.002 |
|  | (0.004) | (0.005) |
| Age | 0.003 | 0.001 |
|  | (0.004) | (0.004) |
| Ethnicity | 0.016 | 0.013 |
|  | (0.046) | (0.046) |
| Number of children | 0.026 | 0.027* |
|  | (0.016) | (0.016) |
| Marital status | 0.275*** | 0.276*** |
|  | (0.092) | (0.092) |
| Self-assessment of health | 0.026*** | 0.026*** |
|  | (0.010) | (0.010) |
| Chronic disease | -0.054 | -0.054 |
|  | (0.037) | (0.037) |
| Family Characteristic | YES | YES |
| Parental Characteristics | YES | YES |
| Spousal Characteristics | YES | YES |
| Provincial fixed effect | YES | YES |
| Survey year fixed effect | YES | YES |
| Observations | 12,758 | 12,758 |
| R^2^ | 0.316 | 0.316 |

Note: The robust standard errors of heteroscedasticity are in parentheses. All the test scores are age-standardized test scores. The family characteristic control variables include family per capita income and family size. The parental characteristics control variables included the following: father's age, father's education, mother's age, and mother's education. The control variables for spousal characteristics included spousal age and spousal education.

* p<0.1; ** p<0.05; *** p<0.01.
